# Stroke Risk Status, Anticoagulation Treatment, and Quality-of-Life in Chinese Patients with Atrial Fibrillation: China Registry of Atrial Fibrillation (CRAF)

**DOI:** 10.1155/2019/7372129

**Published:** 2019-03-21

**Authors:** Yihong Sun, Jun Zhu, Changsheng Ma, Shaowen Liu, Yanzong Yang, Dayi Hu

**Affiliations:** ^1^China-Japan Friendship Hospital, Beijing, China; ^2^Emergency and Intensive Care Center, State Key Laboratory of Cardiovascular Disease, Fuwai Hospital, National Center for Cardiovascular Diseases, Chinese Academy of Medical Sciences and Peking Union Medical College, Beijing 100037, China; ^3^Cardiology Division, Beijing An Zhen Hospital, Capital Medical University, Beijing, China; ^4^Department of Cardiology, Shanghai First People's Hospital Affiliated to Shanghai Jiaotong University, Shanghai 200080, China; ^5^Department of Cardiology, First Affiliated Hospital of Dalian Medical University, Dalian, 116000, China; ^6^Department of Cardiology, Peking University People's Hospital, Beijing, 100044, China

## Abstract

*Objective. *To investigate the contemporary status of stroke risk profile, antithrombotic treatment, and quality-of-life (QoL) of patients with all types of atrial fibrillation (AF) in China.* Design.* This is a multicenter, cross-sectional study.* Setting.* Tertiary (80%) and Tier 2 hospitals (20%) were identified in different economic regions (Northeast, East, West, and Middle) by using a simple random sampling.* Participants. *A total of 3562 (85.6%) patients with nonvalvular atrial fibrillation (NVAF) and 599 (14.4%) with rheumatic valvular atrial fibrillation (VAF) were consecutively enrolled from 111 hospitals from July 2012 to December 2012.* Data Collection.* Patient information was collected and QoL was assessed using Short-Form 36 Health Survey (SF-36) questionnaire.* Primary and Secondary Outcome Measures.* The risk of stroke was assessed using the CHADS_2_ and CHA_2_DS_2_-VASc. QoL was assessed using Medical Outcomes Study SF-36 questionnaire.* Results.* Overall, 31.7% of the patients received anticoagulant treatment and 61.2% received antiplatelet treatment. The rate of anticoagulant treatment was higher in patients with VAF than in those with NVAF. The anticoagulant use was the lowest in Northeast and the highest in Middle regions. Independent risk factors associated with underuse of anticoagulants for NVAF were age, systolic blood pressure (SBP), non-Middle regions, nontertiary hospitals, and new-onset or paroxysmal AF. For VAF patients, the independent factors were age, paroxysmal AF, treatment in Tier 2 hospitals, SBP, diastolic blood pressure, history of coronary artery disease, and nonreceipt of antiarrhythmic therapy. Patients receiving anticoagulants fared significantly better in some QoL domains than those who received no antithrombotic therapy.* Conclusions. *These findings suggest that antiplatelet treatment is overused and anticoagulant treatment is underused both in Chinese patients with VAF and NVAF, even though usage of anticoagulants is associated with better QoL. Risk factors with underuse of anticoagulants were not identical in patients with NVAF and VAF.

## 1. Introduction

Atrial fibrillation (AF) poses a major health problem and economic burden for the countries worldwide. In China, a population survey estimated that there were 4.2 million patients with AF in 2002 [1]. The incidence of AF in China rises sharply from around zero in 30- to 39-year age group to 7.5% in 80- to 89-year age group [1]. Therefore, the incidence is expected to increase markedly with aging in the Chinese population in future decades. As the prevalence of rheumatic valvular disease is relatively higher in low-income countries, the etiology of valvular atrial fibrillation (VAF) in China may be different from that in western countries [2]. In addition, the clinical profile of nonvalvular atrial fibrillation (NVAF) may be different from that in western populations. Currently, only limited data are available on the management of AF in China. A study on racial/ethnic differences in patients with nonrheumatic AF concluded that more Chinese patients suffered stroke than other racial/ethnic groups. Also the risk of intracranial hemorrhage with anticoagulant treatment was higher in Chinese patients [3].

Anticoagulant treatment is pivotal for the prophylaxis of thromboembolic events in patients with AF. Both European and Chinese guidelines recommend that CHADS_2_ or CHA_2_DS_2_-VASc score [4, 5] is used for stratification of the stroke risk, which has been validated in Chinese patients [6, 7]. Despite treatment guideline recommendations on oral anticoagulants, such as warfarin or non-vitamin K antagonist oral anticoagulants (NOACs) to patients with a CHA_2_DS_2_-VASc score ≥2 [8], there are limited data to evaluate guideline adherence in “real world” clinical practice. Recent data on 17,000 patients who were enrolled in the Global Anticoagulant Registry in the Field–Atrial Fibrillation (GARFIELD-AF) study indicated that over 35% of patients with CHA_2_DS_2_-VASc score ≥2 received no anticoagulants, while over 41% of patients with low stroke risk received treatment [9].

Even though one of the major drawbacks of anticoagulant treatment is the risk of bleeding, patients with high risk of bleeding based on HAS-BLED (Hypertension/Abnormal renal and liver function/Stroke/Bleeding/Labile INRs/Elderly/Drug or alcohol use) score have demonstrated a net clinical benefit with warfarin therapy [10]. However, with the risk of stroke, there is very few data on the bleeding risk in Chinese patients with AF. A better understanding regarding the risk of bleeding would be helpful in evaluating the benefits of warfarin versus NOACs. At present, the latter are not reimbursed in China.

In this study, we evaluated the risk of thromboembolism and bleeding in a representative sample of outpatients with AF from different regions of mainland China who were treated at different categories of hospitals. A principal objective of the study was to identify the factors leading to the underuse of anticoagulant treatment and thereby improving the prevention of stroke in Chinese patients.

## 2. Methods

The China Registry of Atrial Fibrillation (CRAF) was a multicenter, observational, cross-sectional registry study. We developed a nationally representative sample of AF patients; 80% of the centers were tertiary hospitals and 20% were Tier 2 hospitals. We identified hospitals using a simple random sampling procedure in each of the economic regions: Northeast, East, West, and Middle regions. From July 2012 to December 2012, patients were consecutively enrolled from 111 hospitals. The approval of independent ethics committees and hospital-based institutional review boards was obtained. The study was conducted in accordance with the principles of Declaration of Helsinki and local regulatory requirements. All enrolled patients provided written informed consent to participate in the study.

### 2.1. Eligibility Criteria

Patients aged 18 years or over with a confirmed diagnosis of either NVAF or VAF were eligible for enrollment in the study. The diagnosis of AF was established either electrocardiographically or by Holter monitoring (AF lasting ≥30 s). Patients with transient reversible causes of AF, including hyperthyroidism, acute pulmonary embolism, recent major surgery, or acute myocardial infarction were excluded. VAF was defined as AF in patients with a history of rheumatic heart disease or mechanic valve replacement; otherwise, patients were defined as having NVAF.

### 2.2. Data Collection

Patient data were collected by interviews, including demographic information, clinical characteristics, medical history (including comorbid cardiovascular disease and bleeding history), the date and method of diagnosis, presence of AF-related symptoms, antithrombotic treatments administered (which included aspirin, clopidogrel, vitamin K antagonists, direct thrombin inhibitors, and factor Xa inhibitors), and the reasons for not administering anticoagulants where applicable. The diagnosis of all medical conditions, including the type of AF, was established from the patients' clinical records. A data collection form was designed and used to record all the information.

All the patients were categorized into 4 types of AF exclusively. Persistent AF was defined as episodes that do not terminate spontaneously, but can be converted with either electrical conversion or pharmacological cardioversion; paroxysmal AF was defined as episodes terminating spontaneously; and permanent AF was defined as episodes that do not terminate either spontaneously with electrical conversion or by pharmacological cardioversion, or where cardioversion had not been attempted. New-onset AF was defined as AF occurring for the first time irrespective of the duration of the arrhythmia or the presence and severity of AF-related symptoms. For patients on oral anticoagulants, international normalized ratio (INR) values over 6 months prior to the enrollment were collected.

CHADS_2_ and CHA_2_DS_2_-VASc scores were used to assess the risk of stroke in all patients, respectively. Heart failure, hypertension (blood pressure ≥140/90 mmHg or antihypertensive treatment), age ≥75 years, diabetes mellitus, and prior stroke or transient ischemic attacks (TIAs) were used to determine, retrospectively, the stroke risk according to CHADS_2_ score. Additionally, a left ventricular ejection fraction <40%, prior thromboembolism, vascular disease (acute coronary syndrome or peripheral artery disease), age 65 to 74 years, and female sex were used to determine the stroke risk according to CHA_2_DS_2_-VASc score. In addition, a HAS-BLED score was also determined to assess the bleeding risk. For the patients who were not taking warfarin, labile INR was omitted in the calculation. Thromboembolic and hemorrhagic events were retrospectively collected. The definition of thromboembolic events included stroke, TIA, or peripheral artery embolism. Hemorrhagic events were defined as clinically significant bleeding that either requires medical intervention to stop or treat bleeding or requires hospitalization, including intracranial bleeding, gastrointestinal bleeding, and other sites bleeding (e.g., epistaxis requiring visit to medical facility for packing).

Quality-of-life (QoL) was assessed by SF-36 (Medical Outcomes Study Short-Form 36 Health Survey) questionnaire at the initial interview. For all patients taking warfarin, INR values, and warfarin dosages over the previous 6 months were also collected. Finally, data on all thromboembolic and bleeding events that occurred were collected, along with the costs of the patients' AF-related treatment.

The registry data were recorded on paper case report forms (CRFs) and then were entered into an electronic database. All data were examined by an independent contract research organization (CRO) to ascertain their completeness and accuracy, and any data queries were forwarded to the participating sites. For each analysis, the data were extracted and analyzed by an independent statistician.

### 2.3. Statistical Analysis

Continuous variables were expressed as means ± standard deviation (SD), and categorical variables as frequencies and percentages. Differences between groups were tested for statistical significance using a chi-squared test for categorical variables and an unpaired* t*-test for continuous variables. Mann-Whitney U test can be applied when the continuous variable is not normally distributed. Logistic regression analysis was used to identify independent risk factors associated with anticoagulant use in patients with both NVAF and VAF. For the multivariable logistic regression analysis, we selected all the variables with p value <0.05 in univariate analysis.

All p values were two sided, and values <0.05 were considered statistically significant. Statistical analyses were performed using SAS® software version 9.1.3 (SAS Institute Inc., Cary, NC, USA).

## 3. Results

### 3.1. Baseline Characteristics

After exclusion of 20 patients because of incomplete information, a total of 4161 patients with confirmed diagnosis of AF were recruited in the study. Most patients (82.2%) were enrolled from tertiary hospitals, with NVAF (85.6%) and VAF (14.4%). Mean age of the patients was 68.3 ± 11.9 years, and 53.1% were male. As shown in Table 1, patients with VAF were more likely to be female and younger than those who had NVAF.

Types of AF were presented in Table 1, where 12.3% of patients had new-onset AF, 32.4% had paroxysmal AF, 31.1% had persistent AF, and 23.9% had permanent AF. More patients with NVAF had new-onset AF (13.2% vs 6.8%) and paroxysmal AF (35.3% vs 15.0%) compared with VAF patients.

### 3.2. Cardiovascular Conditions

Hypertension was present in 57.1% of the patients, while 33.7% had coronary artery disease, 38.2% had congestive heart failure, and 16.9% had diabetes mellitus. More patients with VAF had a history of heart failure than those with NVAF (58.9% vs 34.7%, respectively). However, patients with NVAF were more likely to have both coronary artery disease (37.3% vs 12.4%, respectively) and hypertension (61.8% vs 29.4%, respectively) than those with VAF. Overall, a history of ischemic stroke/TIA and systemic embolism was present in 17.4% of patients, with no significant difference between patients with NVAF and VAF. A history of treatment-related bleeding events was observed in 7.5% of patients, with a higher proportion of VAF patients experiencing bleeding events than NVAF patients (12.9% vs 6.5%, respectively).

Of the 3562 patients, 3551 with NVAF, CHADS_2_, and CHA_2_DS_2_-VASc scores were available to assess the stroke risk. The mean CHADS_2_ score was 1.90 ± 1.46, and 17.9%, 27.7%, and 54.4% of the patients demonstrated a scores of 0, 1, and ≥2, respectively. The mean CHA_2_DS_2_-VASc score was 3.00 ± 1.83, and 8.4%, 15.1%, and 76.6% of the patients demonstrated scores of 0, 1, and ≥2, respectively. The mean HAS-BLED score was 1.66 ± 0.99, and 18.0% of patients demonstrated a score ≥3. In patients with VAF, 40.2% have CHADS_2_ ≥ 2 and 72.8% have CHA2DS2-VASc ≥ 2. Only 10.2% of VAF patients have HAS-BLED scores ≥ 3 (Supplementary Table I).

### 3.3. Rates of Antithrombotic Treatment

Overall, 31.7% of the patient cohort received anticoagulant treatment. Very few patients (n = 37; 0.9%) received NOACs, including 36 cases of rivaroxaban and 1 case of dabigatran. But 61.2% received an antiplatelet agent and 2.0% received an antiplatelet agent combined with an anticoagulant.

**Figure 1:**
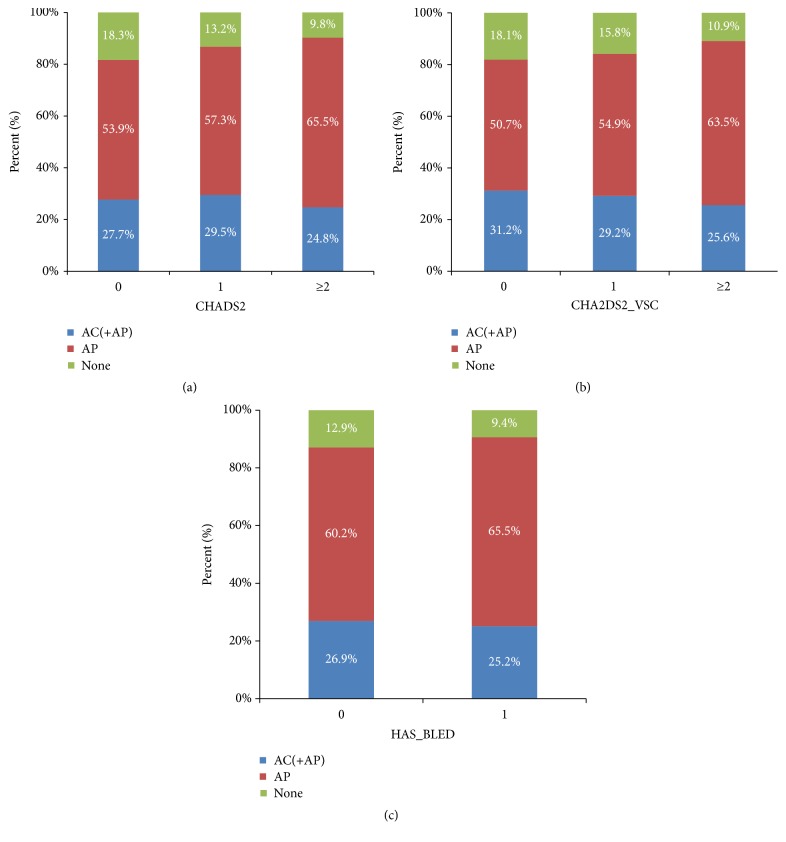
Antithrombotic treatment therapies in patients with nonvalvular atrial fibrillation according to CHADS_2_, CHA_2_DS_2_-VASc, and HAS-BLED scores (AC: anticoagulants; AP: antiplatelet agents).

In patients with NVAF, the most frequently used antithrombotics were antiplatelet agents (61.15%). Figures 1(a) and 1(b) showed the proportions of patients with NVAF receiving antithrombotic treatment according to the stroke risk as assessed by CHADS_2_ (Figure 1(a)) and CHA_2_DS_2_-VASc scores (Figure 1(b)). Results revealed that the use of anticoagulants was decreased with increasing CHADS_2_ or CHA_2_DS_2_-VASc score, where only 24.8% of NVAF patients demonstrated a CHADS_2_ score ≥2, and 25.6%, who received anticoagulants alone or in combination with antiplatelet agents, demonstrated CHA_2_DS_2_-VASc score ≥2. However, 31.2% of patients with NVAF who had a CHA_2_DS_2_-VASc score of 0 received anticoagulants. In terms of bleeding risk, the use of anticoagulants in patients with a HAS-BLED score ≥3 was slightly lower than in those with HAS-BLED scores between 0 and 2 (Figure 1(c)).

The use of anticoagulants in VAF patients was significantly higher than in patients with NVAF (57.3% vs 25.6%, respectively; p < 0.001). However, the use of antiplatelet agents by patients with VAF was lower (168/599; 28.05%). For the patients taking warfarin, we collected the INRs during last 6 months. Overall, the median TTR is 29.2% (12.9%, 51.7%) for those with at least 3 INRs (n = 333).

### 3.4. Factors Associated with Use of Anticoagulants

Univariable analyses of the underuse of anticoagulant in patients with NVAF and VAF were shown in Supplementary Tables 2 and 3. Multivariate logistic regression analysis showed that older patients and those with a higher systolic blood pressure (SBP), new-onset or paroxysmal AF, and treatment in a secondary hospital were less likely to receive an anticoagulant. On the other hand, patients with a history of dyslipidemia, a history of thromboembolism, receipt of antiarrhythmic treatment, and residence in the Middle region of China demonstrated a higher likelihood of receiving an anticoagulant

Independent risk factors associated with underuse of anticoagulants in patients with VAF were older age, lower SBP, higher diastolic blood pressure (DBP), history of coronary artery disease, treatment in a secondary hospital, new-onset or paroxysmal AF, and nonreceipt of antiarrhythmic treatment (Table 2).

### 3.5. Influence of Antithrombotic Treatment on QoL

After adjusting for potential confounding factors, patients with NVAF who received anticoagulants had better physical functioning and emotional role functioning than those who received antiplatelet agents or neither treatment (Table 3). Both anticoagulants and antiplatelet agents were associated with better general health scores. In comparison with patients who received antiplatelet agents, mental health scores were higher in those who received anticoagulants.

Similarly, in patients with VAF, those who received anticoagulants had better physical functioning, social role functioning, and emotional role functioning than those who received antiplatelet agents or neither treatment (Table 3).

## 4. Discussion

In this cross-sectional, nationwide survey, the use of anticoagulants in Chinese patients with CHA_2_DS_2_-VASc scores ≥2 was found to be a slight higher over 50% in those with VAF and a little over 25% in those with NVAF. In addition, the survey found that the use of anticoagulants was decreased as the CHADS_2_ and CHA_2_DS_2_-VASc scores were increased, and there was regional variation in the rate of anticoagulant treatment, with the highest rates being evident in the Middle regions of China. Multivariate logistic regression analysis showed that the independent risk factors associated with underuse of anticoagulants were similar in patients with VAF and NVAF, which included older age, new-onset or paroxysmal AF, treatment in secondary hospitals, and nonreceipt of antiarrhythmic therapy. Knowledge regarding these factors that predict the underuse of anticoagulants is important and necessary for improving the treatment aimed at preventing thromboembolic events in patients with AF.

Rheumatic AF is still an important contributor to AF and accounted for about 14% of patients in this study. Although the proportion with rheumatic AF was lower than the value reported in hospitalized patients some years ago (20%) [1], it is similar to the value reported by the RE-LY Registry (15.7%) [2]. The mean age of patients with VAF in this study was 62 years, and 68.3% were female, which differed significantly from patients with NVAF who were older (mean 69.3 years) and more commonly male (56.7%). In comparison with the Chinese patients in the RE-LY Registry [2], the patients in this cohort were older, and this implies that the incidence of rheumatic valvular disease has been decreased in recent years with the growth of Chinese economy. However, we found that the rate of anticoagulant treatment remains quite low in China. An important reason for this might be due to higher prevalence of rheumatic AF in rural and remote regions of the country, where the availability of health services is much less. We also found that patients who did not receive antiarrhythmic treatment were less likely to be taking anticoagulants, and older age was evidently a common contraindication for their use, a finding that has also been reported in patients with NVAF [11]. In addition, we found that patients with paroxysmal AF were less likely to be prescribed anticoagulants, which may reflect physicians' perceptions of atherosclerosis and the risk of stroke.

In patients with NVAF, hypertension was the most commonly seen comorbid condition. Patients with hypertension have 1.7-fold higher risk of developing AF than normotensive individuals, and 1 in 6 cases of AF has been attributed to hypertension [12, 13]. Hypertension is also one of the major risk factors for thromboembolic events. Hypertension results not only in LV hypertrophy but also in arterial stiffening. Both history of hypertension and the blood pressure are indicators of high risk of stroke [14, 15]. In the ROCKET AF trial (Rivaroxaban Once Daily Oral Direct Factor Xa Inhibition Compared with Vitamin K Antagonism for Prevention of Stroke and Embolism Trial in Atrial Fibrillation), lower screening SBP was associated with a higher risk of vascular death [16]. One Korean study showed that AF patients with BP ≥130/80 mm Hg were at significantly higher risks of major cardiovascular events than patients with BP of 120 to 129/<80 mm Hg in those with oral anticoagulant–naïve and undergoing hypertension treatment [17]. In China, hypertension is the most important predictor for stroke for patients with and without AF [1, 18], and optimal control of blood pressure together with anticoagulant treatment is therefore critical in the prevention of stroke. Moreover, better control of blood pressure could reduce the risk of bleeding related to anticoagulant treatment [10]. As high systolic BP was found to be an independent factor associated with underuse of anticoagulants, it is pivotal for the prevention of stroke in patients with AF as effective strategies are employed to diagnose and control hypertension.

Although the overall rate of anticoagulant use in Chinese patients with NVAF was low compared to western patients, the rate is improving gradually. Compared with the survey that was conducted in 2002 [1], we found that the proportion of Chinese patients not receiving either anticoagulants or antiplatelet agents was lower, although the proportion of patients taking antiplatelet agents remained the highest. In a cohort of NVAF patients studied in Beijing, the rate of anticoagulant use was increased from 30.2% to 57.7% in patients with CHA_2_DS_2_-VASc scores ≥2 over the years 2011 to 2014 [19]. This finding supports our results that there exists large regional variation in the rates of anticoagulant use, which were the lowest in the Northeast region, a less developed area of China. In addition to the regional variation, treatment in secondary hospitals also predicted the underuse of anticoagulants. This indicates that better health policies and academic education are required for the improvement of care in patients with AF in less developed regions.

Our results showed that the use of anticoagulants was decreased with increasing CHA_2_DS_2_-VASc scores which was similar to the findings reported by the GARFIELD Registry study [9]. Current treatment guidelines recommend the use of anticoagulants for all patients with NVAF who have a CHA_2_DS_2_-VASc score ≥2, providing that they do not have any contraindications to anticoagulants. Although only 25.6% of NVAF patients with a CHA_2_DS_2_-VASc score ≥2 received anticoagulants in this study, anticoagulants were overused in 31.2% of patients who had a CHA_2_DS_2_-VASc score of zero. Again, these results provide considerable room for improvement by adhering to current guidelines for anticoagulant treatment of NVAF patients in China.

Patients with new-onset AF and paroxysmal AF accounted for 12.3% and 32.4%, respectively, of our study cohort. Both new-onset AF and paroxysmal AF were significantly associated with underuse of anticoagulants in comparison with patients with persistent or permanent AF, and this finding was consistent with previous studies conducted in both the US [20] and China [19]. Although treatment guidelines recommend the use of anticoagulant treatment for patients with NVAF according to their risk stratification regardless of the type of AF [5, 8], differing results concerning the impact of the AF type on the risk of thromboembolism and death have been reported. While a meta-analysis of randomized, controlled trials and cohort studies showed that nonparoxysmal AF was associated with a higher risk of thromboembolism and death than paroxysmal AF [21], a European registry found that the rate of thromboembolism after 1 year of follow-up showed no difference between patients with paroxysmal and nonparoxysmal AF [22]. In general, patients with paroxysmal AF are younger and have lower stroke risk scores, which may further explain their reduced risk of thromboembolism. However, analysis of patients receiving aspirin indicated that the risks associated with paroxysmal AF are lower than those with nonparoxysmal AF, even at the same stroke risk score [23]. Thus, further studies are needed to clarify whether the type of AF should be included in the stroke risk prediction model.

Around one-third of patients with NVAF in our study had coronary artery disease (CAD), but the optimal antithrombotic treatment for this subgroup lacks solid evidence. We found that history of CAD was an independent risk factor for underuse of anticoagulants in patients with NVAF, possibly reflecting the concerns related to increased risk of bleeding with combination of anticoagulants and antiplatelet agents [24–26]. Although an expert consensus panel [27] has recommended a defined period for the combination therapy of anticoagulant and antiplatelet agent for patients with a recent acute coronary syndrome (ACS) event or percutaneous coronary interventions (PCIs), and an anticoagulant alone for those with stable CAD, determining the optimal strategy for this patient group requires additional studies.

Until now, only sparse data on the QoL of Chinese patients with AF have been reported. Most patients with AF exhibited specific symptoms [28], and both AF-related symptoms and lower QoL were associated with higher risk of hospitalization [29]; hence it is wise to take QoL into account when deciding the treatment. In this regard, warfarin has been criticized because of the frequent INR monitoring requirement, lifestyle and dietary restrictions, fluctuations in dosage, and high risk of bleeding associated with its use, all of which may impact QoL. However, with the use of SF-36 instrument, we found that anticoagulant treatment was associated with better QoL after adjusting for confounding factors. It is postulated that this benefit arises from anticoagulation per se rather than the specific medication administered. This was evidenced by a subgroup analysis of the RE-LY study, which showed no differences in QoL scores between dabigatran and warfarin [30].

The sample size and the selection of study centers that provide geographically contiguous data and treatment at both secondary and tertiary hospitals endow with good evidence regarding the current status of antithrombotic treatment in patients with AF in China. The study also provides valuable information on the clinical profiles of Chinese patients with VAF, data for which was previously very sparse. Limitations of the study include the possibility that the patients selected were not representative of those treated at primary care and community hospitals in China as patients with AF are generally diagnosed and treated at secondary and tertiary hospitals. Also, we did not collect echocardiography data to verify the diagnosis of rheumatic valve disease, and INR and warfarin dose data were collected retrospectively and were incomplete for some patients (although the source data were verified). Another potential limitation is that few patients were taking NOACs as there were no specific indications for the use of either direct thrombin inhibitors or factor Xa inhibitors during the study. Although “real world” data from Taiwan has shown favorable effects of NOACs compared with warfarin in Asian patients [31], clinical experience with NOACs is limited and further evaluation of their use in “real world” clinical practice settings in China is required.

Another issue worth mentioning is that there are considerably more AF patients with rheumatic valvular disease and prosthetic valve replacements in China compared to developed countries. Until recently, dose-adjusted warfarin therapy has been the only effective treatment for the prevention of stroke in these patients.

## 5. Conclusion

This large, nationwide registry of outpatients with VAF and NVAF has confirmed that anticoagulants remain underused in China, despite the finding that anticoagulant treatment is associated with better QoL compared with no antithrombotic treatment. For patients with NVAF, the use of anticoagulants was not in proportion to the risk of stroke, and there was considerable regional variation in the treatment of these patients. Clinical characteristics that could predict the underuse of anticoagulants include older age, CAD, and new-onset AF or paroxysmal AF. These findings indicate that further studies are necessary in specific groups of patients with AF and that further initiatives are warranted to increase the use of anticoagulants in patients with AF.

## Figures and Tables

**Table 1:** Patient characteristics.

Characteristics	Total	NVAF	VAF	p-Value
	(n = 4161)	(n = 3562)	(n = 599)	
Age (years), mean ± SD	68.3 ± 11.9	69.3 ± 11.6	62.0 ± 11.5	<0.001
Female, n (%)	1950 (46.9)	1541 (43.3)	409 (68.3)	<0.001
Duration of AF (years), mean ± SD	5.7 ± 6.9	5.1 ± 6.3	8.5 ± 9.1	<0.001
Region, n, (%)				0.019
Northeast	138 (3.3)	125 (3.5)	13 (2.2)	
East	2664 (64.0)	2279 (64.0)	385 (64.3)	
West	583 (14.0)	480 (13.5)	103 (17.2)	
Middle	776 (18.6)	678 (19.0)	98 (16.4)	
Hospital level, n (%)				0.305
Secondary	742 (17.8)	644 (18.1)	98 (16.4)	
Tertiary	3419 (82.2)	2918 (81.9)	501 (83.6)	
Current smoker, n (%)	411 (9.9)	383 (10.8)	28 (4.7)	<0.001
BMI (kg/m^2^), mean ± SD	24.0 ± 3.5	24.3 ± 3.5	22.7 ± 3.4	<0.001
SBP (mmHg), mean ± SD	129.6 ±18.1	130.7 ±18.0	123.2 ±17.1	<0.001
DBP (mmHg), mean ± SD	78.2 ± 11.6	78.7 ± 11.6	75.2 ± 11.1	<0.001
Type of AF, n (%)				<0.001
New-onset	511 (12.3)	470 (13.2)	41 (6.8)	
Paroxysmal	1347 (32.4)	1257 (35.3)	90 (15.0)	
Persistent	1293 (31.1)	1072 (30.1)	221 (36.9)	
Permanent	995 (23.9)	752 (21.1)	243 (40.6)	
Cardiovascular disease, n (%)				
Coronary artery disease	1395 (33.7)	1321 (37.3)	74 (12.4)	<0.001
Hypertension	2375 (57.1)	2199 (61.8)	176 (29.4)	<0.001
Diabetes mellitus	705 (16.9)	624 (17.5)	81 (13.5)	0.013
Heart failure	1588 (38.2)	1235 (34.7)	353 (58.9)	<0.001
NYHA classification, n (%)				<0.001
Class I	36 (0.9)	30 (0.8)	6 (1.0)	
Class II	397 (9.6)	323 (9.1)	74 (12.4)	
Class III	745 (17.9)	577 (16.2)	168 (28.2)	
Class IV	388 (9.3)	101 (16.9)	577 (16.2)	
Peripheral artery disease	184 (4.4)	161 (4.5)	23 (3.8)	0.441
Renal dysfunction	186 (4.5)	167 (4.7)	19 (3.2)	0.082
Hepatic disease	168 (4.0)	139 (3.9)	29 (4.8)	0.293
Dyslipidemia	851 (20.5)	772 (21.7)	79 (13.2)	<0.001
Thromboembolic events, n (%)	722 (17.4)	620 (17.4)	102 (17.0)	0.808
Ischemic stroke	518 (12.5)	448 (12.6)	70 (13.5)	0.538
TIA	123 (3.0)	104 (2.9)	19 (3.2)	0.738
Non-CNS embolism	42 (1.0)	29 (0.8)	13 (2.2)	0.006
Bleeding events	310 (7.5)	233 (6.5)	77 (12.9)	<0.001
GI bleeding	87 (2.1)	74 (2.1)	13 (2.2)	0.641
ICH	67 (1.6)	56 (1.6)	11 (1.8)	0.884
Other sites	156 (3.7)	103 (2.9)	53 (8.8)	<0.001
Antiarrhythmic treatment, n (%)				<0.001
Rhythm control	854 (20.5)	790 (22.2)	64 (10.7)	
Rate control	2330 (56.1)	1899 (53.4)	431 (72.1)	
Both	410 (9.9)	370 (10.4)	40 (6.7)	

AF: atrial fibrillation; BMI: body mass index; CNS: central nervous system; DBP: diastolic blood pressure; NVAF: non-valvular atrial fibrillation; SBP: systolic blood pressure; DBP: diastolic blood pressure; SD: standard deviation; TIA: transient ischemic attack; VAF: valvular atrial fibrillation; GI: gastrointestinal bleeding; ICH: intracranial bleeding.

**Table 2:** Risk factors for underuse of anticoagulants in patients with moderate-to-high risk NVAF and patients with VAF.

Risk factors	NVAF			VAF		
	OR	95% CI	p-Value	OR	95% CI	p-Value
Age (per 10 years)	1.122	(1.040, 1.210)	0.003	1.517	(1.277, 1.802)	<0.001
SBP (per 10 mmHg)	1.102	(1.051, 1.155)	<0.001	0.855	(0.744, 0.982)	0.026
DBP (per 10 mmHg)				1.356	(1.106, 1.662)	0.003
Regions (vs middle)						
Northeast	1.900	1.164, 3.101	0.010			
East	1.438	1.176, 1.760	<0.001			
West	1.270	0.969, 1.665	0.083			
Hospital level (vs tertiary)	0.420	0.324, 0.543	<0.001	0.539	(0.335, 0.865)	0.010
Type of AF (vs persistent/permanent)						
New-onset	1.857	1.412, 2.441	<0.001	1.978	(0.975, 4.013)	0.059
Paroxysmal	1.768	1.470, 2.128	<0.001	1.761	(1.072, 2.895)	0.026
Dyslipidemia	0.718	0.591, 0.873	0.001			
History of CAD				0.198	(0.061, 0.644)	0.007
History of thromboembolism	0.640	0.520, 0.789	<0.001			
Antiarrhythmic treatment	0.359	0.271, 0.475	<0.001	0.461	(0.258, 0.823)	0.009

AF: atrial fibrillation; CAD: coronary artery disease; CI: confidence interval; DBP: diastolic blood pressure; NVAF: non-valvular atrial fibrillation; OR: odds ratio; SBP: systolic blood pressure; VAF: valvular atrial fibrillation.

**Table 3:** The impact of anticoagulant and antiplatelet treatment on QoL assessed by the SF-36 questionnaire in patients with NVAF and VAF (mean scores and 95% CIs).

Domains	Anticoagulants	Antiplatelet Agents	No antithrombotic therapy	p-Value*∗*
*Patients with NVAF*				
Physical functioning	57.29 (53.45, 61.13)^a,b^	54.72 (50.94, 58.50)	53.71 (49.57, 57.85)	0.005
Physical role functioning	34.87 (27.48, 42.25)	32.03 (24.76, 39.30)	33.29 (25.33, 41.26)	0.2601
Bodily pain	72.55 (68.92, 76.18)	71.84 (68.27, 75.41)	73.60 (69.69, 77.51)	0.271
General health	41.76 (38.34, 45.18)^a^	42.18 (38.82, 45.54)^b^	38.40 (34.72, 42.09)	0.002
Vitality	61.54 (58.44, 64.64)	60.97 (57.93, 64.02)	59.13 (55.79, 62.47)	0.070
Social role functioning	59.57 (55.77, 63.38)	59.69 (55.94, 63.43)	58.67 (54.57, 62.78)	0.693
Emotional role functioning	59.35 (51.72, 66.98)^a^	54.74 (47.23, 62.24)^b^	54.41 (46.19, 62.63)	0.026
Mental health	67.92 (65.19, 70.65)^a^	66.21 (63.53, 68.89)	66.11 (63.11, 69.12)	0.040
*Patients with VAF*				
Physical functioning	43.70 (35.79, 51.61)^a,b^	37.96 (29.58, 46.34)	34.69 (25.09, 44.29)	0.004
Physical role functioning	28.86 (10.57, 47.15)	20.99 (2.00, 39.98)	19.02 (−1.04, 39.08)	0.055
Bodily pain	59.92 (53.44, 66.40)	58.86 (51.91, 65.81)	54.46 (46.11, 62.80)	0.231
General health	36.27 (27.50, 45.04)	34.71 (25.60, 43.82)	31.96 (22.34, 41.59)	0.252
Vitality	46.61 (40.32, 52.90)	45.29 (38.62, 51.96)	40.54 (32.90, 48.18)	0.074
Social role functioning	49.44 (41.63, 57.25)^a,b^	45.06 (36.78, 53.33)	42.46 (32.98, 51.94)	0.036
Emotional role functioning	44.51 (31.73, 57.30)^a^	27.60 (13.90, 41.29)	35.68 (19.22, 52.14)	0.001
Mental health	53.25 (47.11, 59.39)	52.88 (46.38, 59.39)	49.02 (41.60, 56.44)	0.276

NVAF: non-valvular atrial fibrillation; QoL: quality of life; SF-36: Medical Outcomes Study Short-Form 36 Health Survey; VAF: valvular atrial fibrillation.

*∗* means p-values referring to differences among the 3 groups.

“a” indicates significant difference versus patients receiving antiplatelet drugs, and “b” indicates significant difference versus patients not on antithrombotic therapy.

## Data Availability

The data used to support the findings of this study are available from the corresponding author upon request.

## Additional Points

*CRAF Investigators*. Bao Li, Baopeng Tang, Bei Shi, Bo Yang, Bo Yu, Chuanyu Gao, Chun Wang, Chunlai Zhang, Dayi Hu, Fang Peng, Farong Shen, Guoan Sheng, Guohong Li, Guoqing Gu, Hanhua Ji, He Huang, Hengliang Liu, Hongliang Cong, Hua Mao, Huaimin Guan, Hui Gong, Hui Song, Huimin Gu, Ji Yan, Jian Qiu, Jianhong Tao, Jianhua Zhu, Jianjun Bai, Jianqiu Liang, Jianyan Dong, Jingquan Zhong, Jingshu Guan, Juan Wang, Jun Zhang (Hebei), Jun Zhang (Pingdingshan), Kui Hong, Li Jiang, Li Xue, Licheng Yu, Lin Pi, Ling Li, Menghua Chen, Mingcai Song, Mingsheng Wang, Pengfei Yu, Penglin Yang, Pingshuan Dong, Quan Fang, Qunying Huang, Ruihua Yue, Shangming Song, Shaohong Dong, Shaowen Liu, Shenwen Fu, Shian Huang, Shouyan Zhang, Shubin Jiang, Shuixiang Yang, Shulin Wu, Shuren Ma, Suxin Luo, Tianlun Yang, Tongguo Wu, Wei Cui, Wei Wang, Xi Su, Xia Mei, Xiangdong Xu, Xiangjun Yang, Xiangyang Ye, Xianlin Ma, Xiaodong Pu, Xiaohua Guo, Xiaoli Yang, Xiaomin Chen, Xiaoping Chen, Xiaoqun Wan, Xiaoqun Zheng, Xin Xu, Xiuli Li, Xudong Yu, Xuekun Zhang, Yafei Yan, Yaling Han, Yan Wang, Yang Zheng, Yanmin Yang, Yanqin Sun, Yansheng Ding, Yihong Luo, Yihong Sun, Ying Wang, Yinglong Hou, Yong Peng, Yong Wang, Yueguang Chen, Yuehui Yin, Yuhua Liao, Yulin Zhang, Yuming Zhang, Yunzhe Tan, Yuyin Luo, Zeqi Zheng, Zhan Lv, Zhanbin Feng, Zheng Wang, ZhenshunXiu, Zhimin Jin, Zhirong Wang, Zhiyuan Song, Zhong Wu, Zhongcai Fan, Zuyi Yuan.

## Supplementary Materials

Supplementary Materials S1 described the distribution of CHADS2, CHA2DS2-VASc, and HAS-BLED scores in patients with VAF. S2 and S3 showed the factors related to the underuse of OAC in patients with NVAF and VAF, respectively.
