# Supplementary material for: Stroke Risk Status, Anticoagulation Treatment, and Quality-of-Life in Chinese Patients with Atrial Fibrillation: China Registry of Atrial Fibrillation (CRAF)
Source: Cardiovasc Ther. 2019 Mar 21;2019:7372129. doi: 10.1155/2019/7372129 (PMC6739760; doi:10.1155/2019/7372129)
Supplement: Supplementary Materials — S1 described the distribution of CHADS2, CHA2DS2-VASc, and HAS-BLED scores in patients with VAF. S2 and S3 showed the factors related to the underuse of OAC in patients with NVAF and VAF, respectively. [file 7372129.f1.pdf]

Supplements

S1 The CHADS2, CHA2DS2-VASc, and HAS-BLED scores in patients with VAF

	N	%
CHADS2 score	599	
0	133	22.2
1	225	37.6
2	114	19.0
3	80	13.4
4	29	4.8
5	16	2.7
6	1	0.2
7	1	0.2
CHA_2DS_2-VSC		
0	36	6.0
1	127	21.2
2	166	27.7
3	106	17.7
4	80	13.4
5	52	8.7
6	21	3.5
7	10	1.7
8	1	0.2

HAS_BLED		
0	218	36.4
1	189	31.6
2	131	21.9
3	49	8.2
4	11	1.8
5	1	0.2

S2 The univariable analysis of the factors associated with under-anticoagulation in patients with NVAF

Characteristics	OAC (n =948)	Non OAC (n = 2614)	p-Value
Age (years), mean ± SD	67.14 ± 11.29	70.12 ± 11.66	<0.001
Female, n (%)	409 (43.1)	1132 (43.3)	0.9313
Duration of AF (years), mean ± SD	5.04 ±5.96	5.15 ±6.42	0.5908
Region, n, (%)			<0.001
Northeast	27 (2.8)	98 (3.7)	
East	543 (57.3)	1736 (66.4)	
West	143 (15.1)	337 (12.9)	
Middle	235 (24.8)	443 (16.9)	
Hospital level, n (%)			<0.001
Secondary	84 (8.9)	560 (21.4)	
Tertiary	864 (91.2)	2054 (78.5)	
Current smoker, n (%)	90 (9.6)	293 (11.3)	0.3014
BMI (kg/m^2), mean ± SD	24.49 ± 3.35	24.17 ± 3.53	0.0114
SBP (mmHg), mean ± SD	127.84 ±16.86	131.77 ±18.30	<0.001
DBP (mmHg), mean ± SD	78.20 ± 10.98	78.84 ± 11.81	0.1274
Type of AF, n (%)			<0.001
New-onset	89 (9.4)	381 (14.6)	
Paroxysmal	293 (31.1)	964 (37.0)	

Persistent	332 (35.2)	740 (28.4)	
Permanent	229 (24.3)	523 (20.1)	
Cardiovascular disease, n (%)			
Coronary artery disease	225 (23.8)	1096 (42.3)	<0.001
Hypertension	565(59.6)	1634 (62.6)	0.1063
Diabetes mellitus	165 (17.4)	459 (17.6)	0.9175
Heart failure	281 (29.7)	954 (36.6)	<0.001
NYHA classification			<0.001
Ⅱ	98(10.3%)	225(8.6%)	
Ⅲ	127(13.4%)	450(17.3%)	
Ⅳ	41(4.3%)	246(9.4%)	
Peripheral artery disease	46 (4.9)	115 (4.4)	0.5642
Renal dysfunction	27 (2.8)	140 (5.4)	0.0010
Hepatic disease	31 (3.3)	108 (4.1)	0.2306
Dyslipidemia	234 (24.7)	538 (20.6)	0.0088
Thromboembolic events, n (%)	198 (20.9)	422 (16.2)	0.0013
Ischemic stroke	138 (14.6)	310 (11.9)	0.0342
TIA	31 (3.3)	73 (2.8)	0.4598
Non-CNS embolism	16 (1.7)	13 (0.5)	0.0011
Bleeding events	83 (8.8)	150 (5.7)	0.0018
GI bleeding	20(2.1)	54(2.1)	0.9354
ICH	19(2.0)	37(1.4)	0.2235

Antiarrhythmic treatment, n (%)			<0.001
Rhythm control	265 (28.0)	525 (20.1)	
Rate control	503 (53.1)	1396 (53.5)	
Both	113 (11.9)	257 (9.8)	

S3 The univariable analysis of the factors associated with under-anticoagulation in patients with VAF

Characteristics	OAC (n =345)	Non OAC (n = 254)	P
Age (years), mean ± SD	60.03 ± 11.6	64.59 ± 11.48	<0.001
Female, n (%)	236 (68.4)	173 (68.1)	0.939
Duration of AF (years), mean ± SD	8.75 ±9.02	8.15 ±9.12	0.4429
Region, n, (%)			0.0130
Northeast	5 (1.4)	8 (2.2)	
East	213 (61.7)	172 (67.7)	
West	67 (19.4)	36 (14.2)	
Middle	60 (17.4)	38 (15.0)	
Hospital level, n (%)			0.009
Secondary	44 (12.8)	54 (21.3)	
Tertiary	301 (87.2)	200 (78.7)	
Current smoker, n (%)	16 (4.7)	12 (4.7)	0.994
BMI (kg/m^2), mean ± SD	22.55 ± 3.20	22.97 ± 3.65	0.052
SBP (mmHg), mean ± SD	122.63 ±16.52	124.03 ±17.84	0.324
DBP (mmHg), mean ± SD	74.16 ± 10.51	76.56 ± 11.79	0.011
Type of AF, n (%)			0.038
New-onset	19 (5.6)	22 (8.7)	
Paroxysmal	42 (12.3)	48 (19.0)	

Persistent	131 (38.3)	90 (36.6)	
Permanent	150 (43.9)	93 (36.8)	
Cardiovascular disease, n (%)			
Coronary artery disease	36 (10.4)	38 (15.1)	0.091
Hypertension	94 (27.2)	84 (32.3)	0.182
Diabetes mellitus	48 (13.9)	33 (13.0)	0.744
Heart failure	211 (61.5)	142 (56.1)	0.186
NYHA classification			<0.001
Ⅱ	58(16.9%)	16(6.3%)	
Ⅲ	103 30.0%)	65(25.7%)	
Ⅳ	43(12.5%)	58(22.9%)	
Peripheral artery disease	18 (5.2)	5 (2.0)	0.441
Renal dysfunction	11 (3.2)	8 (3.1)	0.979
Hepatic disease	16 (4.6)	13 (5.1)	0.787
Dyslipidemia	40 (11.6)	39 (15.4)	0.181
Thromboembolic events, n (%)	64 (18.6)	38 (15.0)	0.246
Ischemic stroke	47 (13.6)	23 (9.1)	0.082
TIA	13 (3.8)	6 (2.4)	0.325
Non-CNS embolism	10 (2.9)	3 (1.2)	0.139
Bleeding events	50 (14.5)	27 (10.6)	0.159
GI bleeding	5(1.4)	8(3.1)	0.161
ICH	9(2.6)	2(0.8)	0.129

others			
Antiarrhythmic treatment, n (%)			0.007
Rhythm control	29 (8.4)	35 (13.8)	
Rate control	265 (77.0)	166 (65.4)	
Both	23 (6.7)	17 (6.7)	
